# Diet Diversity in Carnivorous Terebrid Snails Is Tied to the Presence and Absence of a Venom Gland

**DOI:** 10.3390/toxins13020108

**Published:** 2021-02-02

**Authors:** Juliette Gorson, Giulia Fassio, Emily S. Lau, Mandë Holford

**Affiliations:** 1 Department of Chemistry, Hunter College Belfer Research Center, City University of New York, New York, NY 10021, USA; jmgorson@gmail.com (J.G.); giulia.fassio@uniroma1.it (G.F.); emily.lau@lifesci.ucsb.edu (E.S.L.); 2 Graduate Programs in Biology, Biochemistry, Chemistry, Graduate Center, City University of New York, New York, NY 10016, USA; 3 Division of Invertebrate Zoology, The American Museum of Natural History, New York, NY 10024, USA; 4 Department of Biology, Hofstra University, Hempstead, NY 11549, USA; 5 Department of Biology and Biotechnologies “Charles Darwin”, Sapienza University of Rome, I-00185 Rome, Italy; 6 Department of Biology and Evolution of Marine Organisms (BEOM), Stazione Zoologica Anton Dohrn, I-00198 Rome, Italy; 7 Department of Ecology, Evolution, and Marine Biology, University of California Santa Barbara, Santa Barbara, CA 93106, USA

**Keywords:** diet diversity, venom, Terebridae

## Abstract

Predator-prey interactions are thought to play a driving role in animal evolution, especially for groups that have developed venom as their predatory strategy. However, how the diet of venomous animals influences the composition of venom arsenals remains uncertain. Two prevailing hypotheses to explain the relationship between diet and venom composition focus on prey preference and the types of compounds in venom, and a positive correlation between dietary breadth and the number of compounds in venom. Here, we examined venom complexity, phylogenetic relationship, collection depth, and biogeography of the Terebridae (auger snails) to determine if repeated innovations in terebrid foregut anatomy and venom composition correspond to diet variation. We performed the first molecular study of the diet of terebrid marine snails by metabarcoding the gut content of 71 terebrid specimens from 17 species. Our results suggest that the presence or absence of a venom gland is strongly correlated with dietary breadth. Specifically, terebrid species without a venom gland displayed greater diversity in their diet. Additionally, we propose a revision of the definition of venom complexity in conoidean snails to more accurately capture the breadth of ecological influences. These findings suggest that prey diet is an important factor in terebrid venom evolution and diversification and further investigations of other understudied organisms, like terebrids, are needed to develop robust hypotheses in this area.

## 1. Introduction

Venomous animals use their toxin arsenal for several ecological activities including predation, defense, space competition, and intraspecific communication [1]. In the predator–prey arms-race, diet is widely accepted as a major force in shaping venom evolution across different taxa such as scorpions, snakes, and spiders [2,3,4]. However, there have been few multi-species studies that specifically look at the role of diet on venom composition and complexity [5,6]. Recently, dietary breadth of venomous snails in the family Conidae was positively linked to venom complexity (number of compounds in venom), but not venom composition (type of compounds found in venom) [7]. Such results give credence to a possible association between venom evolution and prey acquisition throughout the animal kingdom [8,9]. Venom, a cocktail of toxic compounds that includes peptides, proteins, and small molecules, is produced to elicit a reaction with targeted organisms. Since venom is fundamentally a molecular trait, the correlation between venom composition and prey phenotype can be used to trace adaptive evolution and ecological processes such as predation and diet [1,10].

There are primarily two current hypotheses to explain the relationship between a venomous predator’s diet and the nature of its venom: (1) prey preference can determine venom components (types of compounds in venom) [4], and (2) dietary breadth and venom complexity (number of compounds in venom) are positively correlated [11,12]. Studies showing that venoms from different species are most effective on their preferred prey help support the first hypothesis that prey preference can drive venom composition [13,14]. Similarly, to support the second hypothesis (the number of compounds in venom), studies have shown that increased prey breadth is correlated with venom complexity. Examples supporting each hypothesis include findings by Phuong et al. (2016) that cone snails with a more generalized diet tend to have more complex venoms [7] and Li et al. (2005) who found a 50- to 100-fold decrease in toxicity of venom in a group of sea snakes that have shifted to eating only fish eggs [7,11]. Knowledge of selective forces, such as diet, that can drive venom diversification is crucial to understanding patterns in venom evolution.

A working theory in venom evolution is that the acquisition of a venom apparatus and the capacity to synthesize venom and deliver venom to prey has led to diversification within clades of venomous organisms. The acquired novelty of venom then presents opportunities to feed on more or different types of prey, thereby opening new niches and facilitating speciation [15,16]. In contrast, we recently found no correlation between the presence of a venom gland and increased diversification rates within the Terebridae (auger snails) [17]. Terebrids are an understudied family of carnivorous marine snails whose structural variations in the foregut anatomy denote a variety of prey capture techniques. In this study, we address the hypothesis that repeated innovations in terebrid foregut anatomy and in their venom composition may correspond to variation in the diet of terebrids. [18,19]. Similar to cone snails, certain species of terebrids produce a complex mixture of peptide and protein toxins in their venom to subdue their annelid prey [20,21,22,23]. Several members of the Terebridae deliver their toxin payload using a venom apparatus comprised of a venom gland, proboscis, and radula sac, similar to the venom apparatuses found in cone snails. Although the ancestral state for terebrid anatomy included a venom apparatus, many lineages of terebrids have since lost a structured venom apparatus and we recently reported twelve distinct terebrid foregut anatomies, only five of which have a traditional venom gland [17]. Those species that lost the structures of the venom apparatus may still possess other accessory organs, such as salivary glands and proboscis feeding structures [17]. Our previous work also found that each foregut type could be found across multiple clades of the terebrid phylogeny [17].

**Figure 1:**
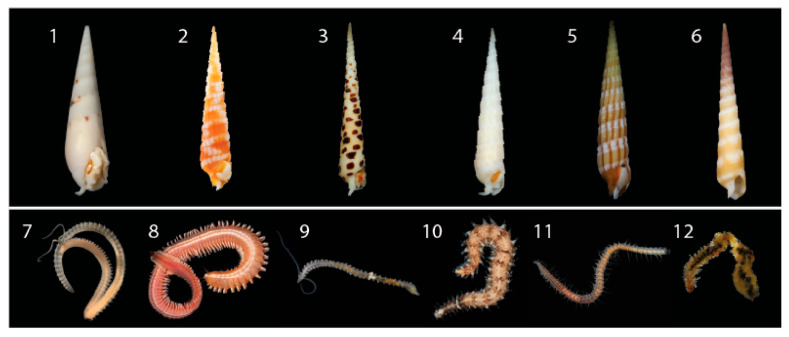
Representative venomous terebrid snails and their annelid prey identified in this study. (**1**) *Hastula hectica*. (**2**) *Myurella nebulosa.* (**3**) *Terebra subulata*. (**4**) *Terebra argus.* (**5**) *Myruellopsis undulata*. (**6**) *Terebra guttata*. (**7**) *Scolelepis.* (**8**) *Neanthes*. (**9**) *Pygospio*. (**10**) *Odontosyllis*. (**11**) *Syllis.* (**12**) Worm pulled from the mouth of *Terebra guttata* (**6**), identified as *Scolelepis*. Photo credits available online: https://www.aphotomarine.com (accessed on 29 January 2021).

Here, we examine the correlation between terebrid foregut anatomies (that imply the presence or absence of a venom apparatus), venom complexity, and prey diversity, by metabarcoding the gut contents of 71 specimens sampled across 17 terebrid species (Figure 1, Supplemental Table S1). The terebrid species selected span eight terebrid genera, belong to five phylogenetic clades (clades B, C, D, E, and F) out of the total six found in the family, and are representative of at least four of the twelve known terebrid foregut anatomies [17]. We used gut and venom gland transcriptomes from each of the 71 terebrid specimens to identify the annelid worm meal, the corresponding venom composition, and to examine the role of diet in the diversity of terebrid venom apparatus and venom composition.

## 2. Results

### 2.1. Terebrid Gut Content Reveals Diverse Annelid Prey

**Figure 2:**
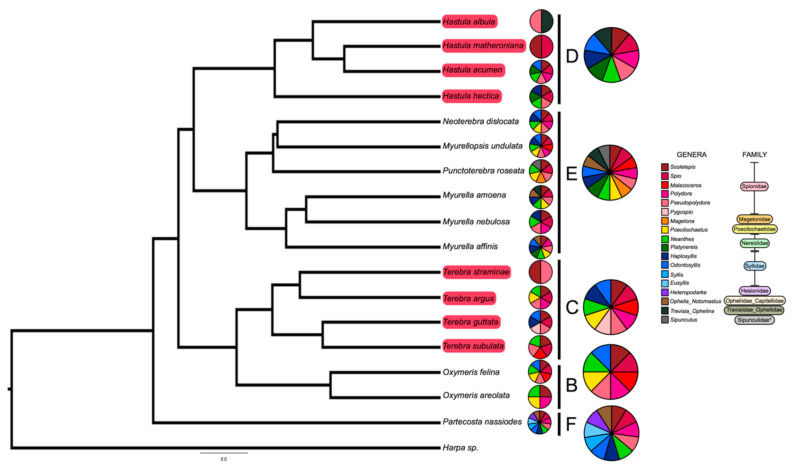
Eighteen different annelid prey genera found in gut of terebrid snails. Shown is the phylogeny of terebrid species used in this study and the annelid worm meal identified in the gut of terebrid specimens. Annelid meals were identified for terebrid species with a venom gland and for those without a venom gland. Terebrid species with a traditional venom gland are highlighted in red in the tree. Smaller pie charts represent the diet of each adjacent taxon. Larger pie charts represent the aggregate diet for terebrids in the same clade. The phylogeny is adapted from the work of Modica et al., 2020, the most up-to-date and first dated phylogeny of the Terebridae family [17].

We sequenced the gut content of 17 snail species, belonging to 5 terebrid clades (clades B, C, D, E, and F). The bioinformatic pipeline firstly isolated a total of 254 prey reads (Table S2). After merging data from species replicates, we obtained 217 unique record indicating the feeding of one snail species on one prey genus (Tables S1 and S2). The range of worm prey genera detected in each terebrid specimen was between 1 and 7 (M = 3.06, SD = 1.37). Worm meals were identified in terebrid lineages with and without a venom apparatus. The range of worm prey genera detected for each terebrid species was between 2 and 11 (M = 6.18, SD = 2.58) (Figure 2).

Overall, eighteen different worm genera, belonging to nine families and two phyla (Anellida and Sipuncula) were identified as terebrid prey. The most represented annelid prey was from the genus *Scolelepis* Blainville, 1828 (Spionidae: Polychaeta), which was found in 16 species out of 17 terebrid species and in 89% of the analyzed specimens (*n* = 71). Three other annelid genera, *Pseudopolydora* Czerniavsky, 1881 (Spionidae: Polychaeta), *Spio* Fabricius, 1785 (Spionidae: Polychaeta) and *Neanthes* Kinberg, 1865 (Nereididae: Polychaeta) were also largely represented in our dataset and were found, respectively, in 13–15 terebrid species and 30–48% of our specimens. DNA of each of these four prey genera were detected in terebrid species collected in all macro sampling areas (Florida, UAE, and Papua New Guinea), in terebrids with and without a venom apparatus, and belonging to five phylogenetic clades investigated. In general, the Annelid genera found as terebrid prey belonged to two classes: Errantia (7 genera) and Sedentaria (10 genera). In all specimens but one, we found at least one Sedentaria prey and all other samples had between 1 and 5 Sedentaria prey genera, while in only half of the dataset (*n* = 36) we detected at least one Errantia prey, with the highest number being 4 (Figure 2). Additionally, a live *Terebra guttata* sample (KVG_256), collected in Papua New Guinea, was found with a worm in its mouth (Figure 1). We sequenced the 16S rDNA gene of the worm (GenBank accession number MW013507) and BLASTed it against the non-redundant protein sequences database. This approach identified the annelid species as *Scolelepis eltaninae* with a reported e-value of 7 × 10^−94^. *S. eltaninae* belongs to the Spionidae family, which was highly prevalent in all terebrid species analyzed in this study. Su

### 2.2. Strong Correlation between Presence and Absence of a Venom Apparatus and Terebrid Diet Diversity

**Figure 3:**
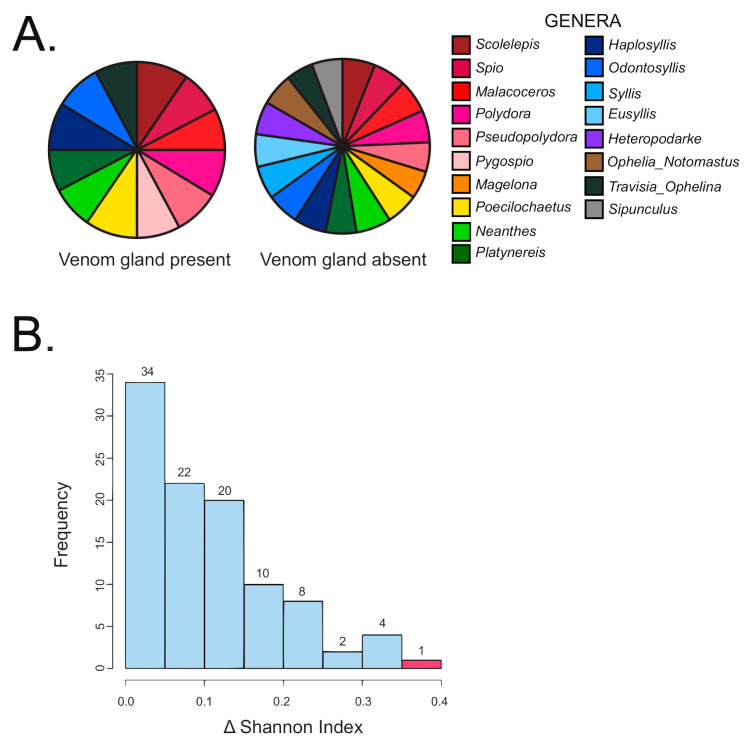
Terebrid snails without a venom gland (VG) have a larger diversity in prey diet. (**A**) Diet diversity in terebrids with and without a VG. (**B**) Histogram showing the frequency of Shannon index differences obtained from Kruskal Wallis non-parametric test on data categorized by venom apparatus (randomized data in blue and study data in red).

The Kruskal Wallis non-parametric test was performed on all 71 specimens and the result indicated a significant difference of diet breadth between terebrid specimens with and without venom apparatus (*p* < 0.01). Specifically, terebrid specimens with a venom gland present had a more restricted diet (12 annelid genera recorded) than terebrid species without a venom gland (16 annelid genera recorded) (Figure 3A). A randomization test of all 71 specimens was conducted to further corroborate the results of the Kruskal Wallis non-parametric test. The difference between the Shannon diversity indices observed for the samples with the presence (1.91) and absence of a venom apparatus (2.26) was calculated and found to be 0.352. Observations were then randomized by column 100 times and the differences in Shannon indices were recalculated. Our data produced the greatest difference in Shannon index (*p* < 0.01), supporting the finding that terebrid species without a venom gland had a greater diversity of annelid diet (Figure 3B).

### 2.3. No Correlation between Terebrid Venom Complexity and Diet Diversity

**Figure 4:**
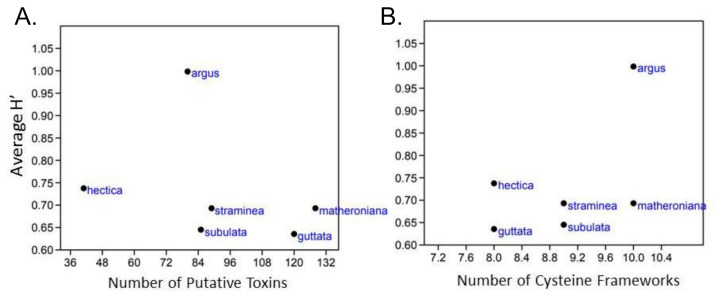
No direct correlation between terebrid diet and venom complexity. Diet breadth, calculated as the Shannon’s diversity index (H’), was plotted against the number of putative toxins (**A**) and with the number of cysteine frameworks (**B**) in six terebrids. No correlation was detected. Analyses were performed with Past3.

The transcriptomes from six terebrid specimens (*Hastula hectica, Hastula matheroniana, Terebra subulata, Terebra guttata, Terebra argus, and Terebra straminea*) were compared with the results of their gut content eDNA to investigate correlations between terebrid venom complexity and diet diversity, where venom complexity was defined by the number of total putative terebrid toxins and the number of cysteine frameworks identified. The number of mature terebrid venom peptide toxins (teretoxins) was quite variable, ranging from 41 to 128 in our analyzed species (Figure 4A).

A small variation in the number of cysteine frameworks, ranging from 8 to 10 cysteines, was instead detected among the examined terebrid species (Figure 4B). *Hastula hectica* showed the lowest venom complexity while *Hastula matheroniana* showed the highest. Plots of the average species Shannon index against the number of teretoxins and cysteine frameworks did not produce any quantitative statistical results, indicating there is no direct correlation between terebrid venom complexity and diet (Figure 4).

**Figure 5:**
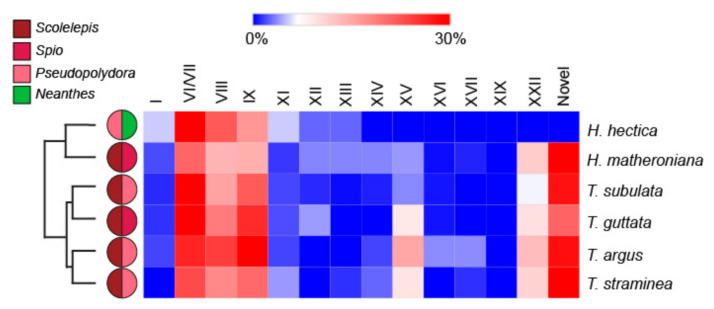
Terebrid species with different diet produce similar venom toxins. Heat map showing the interspecific variation of relative cysteine frameworks in terebrid toxins. Adjacent pie charts represent the identified diet for each specimen. Most of the specimens show similar relative percentages of each cysteine frameworks but *H. hectica* has higher values for frameworks I and XI, and no framework XXII putative teretoxins found in all other terebrid samples use in this study.

However, there are qualitative results worth noting. For example, *Hastula hectica* has a venom composition different from the other five individuals analyzed in this study (Figure 5).

Specifically, *H. hectica* has a higher proportion of frameworks I and XI putative teretoxins and surprisingly has no framework XXII putative teretoxins, which on average account for approximately 7% of all teretoxins found in all specimens tested. Interestingly, of the six individuals analyzed, *H. hectica* was the only individual that consumed *Neanthes* and did not consume *Scolelepis,* which was found as prey in all other terebrid species examined (Figure 5). Likewise, *T. subulata, T. argus,* and *T. straminea* all consume the same genera of annelids (*Scolelepis* and *Pseudopolydora*) but their venom composition is not the same. A similar pattern is shown with *H. matheroniana* and *T. guttata*, further suggesting venom complexity may not be associated with diet preferences.

### 2.4. No Correlation between Shell Length and Diet Diversity

To investigate other factors of terebrid diversification that might not be tied to venom, we investigated abiotic characters pertaining to shell length, geographic location, and depth using all 71 specimens. No correlation emerged between shell length and number of prey genera for each specimen (Figure S1B). The Jaccard heat map identified similarity in diet between *Myurella undulata* and *Terebra dislocata*. However, Shannon’s diversity index (H’) distributions showed nonsignificant differences between phylogenetic clades, macro collecting areas, and shell lengths (Figure S1A). In addition, no significant differences were also detected when comparing H’ distributions and maximum, minimum, average and range of collection depth. These findings may suggest abiotic pressures may have a limited role in terebrid annelid diet diversity.

## 3. Discussion

How the diet of venomous animals influences the composition of venom arsenals is an ongoing debate [7,17,24]. Elucidation of the selective forces that drive diversification of venomous organisms, requires examination of variations in mechanisms of delivery (beaks, fangs, harpoons, spines, etc.), disparities in venom composition (proteins, peptides, small molecules, etc.), and prey preferences. The degree of anatomical variation found in the foregut of terebrids, including the presence or absence of a comprehensive venom delivery apparatus, was previously thought to be correlated with venom composition and annelid prey preference [18,25,26]. We examined this hypothesis using a next generation sequencing approach, molecularly identifying (for the first time) prey genera in the gut contents of terebrids. We identified a strong correlation between the presence and absence of a venom apparatus and the diversity of the terebrid diet.

In this study, we detected a total of 18 annelid prey genera being consumed by terebrids, of which *Scolelepis* and *Spio* (both in the Spionidae family) were the most prevalent (Figure 1 and Figure 2). Using these data, we found terebrid species with a venom apparatus consume fewer annelid prey types than those without a venom apparatus. Our results were further corroborated with a randomization test and are not an artifact of the sampling group. Our results correspond with the current literature that evolution of venom production can cause prey specificity [7,9,27,28]. In a recent study using snakes, it was found that venom diversity was a result of adaptations to specific diets and the complexity of venom in snakes is due to the fact that venoms are under strong natural selection pressures to combat selection pressures in prey molecular targets [4,27,29]. As with snakes, the qualitative venom variation observed in terebrids with a venom gland (specifically *Hastula hectica*) might be explained by an evolutionary arms race between a terebrid predator and a specific annelid prey, however, additional taxa are needed to corroborate this finding for terebrids.

While the diversity of foregut anatomies found in the entire Conoidean superfamily is present in just the Terebridae family, contrary to what was found for cone snails, we did not find a statistical correlation between venom complexity and diet diversity [7,30]. The lack of statistical significance may reflect: (1) the small sample size (only 6 of the 17 terebrid species were used in the venom variation analysis) and (2) a need to redefine venom complexity. In Phuong et al.’s paper (2016), it was determined that total number of mature toxins was the best indicator of venom complexity, while the number of gene superfamilies and the number of cysteine frameworks were less ideal indicators [7]. Here we used both the total number of mature teretoxins and the diversity of cysteine frameworks to determine venom complexity but did not find a statistical correlation. Although there was no statistical correlation between the genera of prey consumed and the distribution of cysteine frameworks in the venom, there is a qualitative relationship wherein *Hastula hectica* had a different suite of putative teretoxins than the other terebrids analyzed. *H. hectica* was the only terebrid found to consume *Neanthes* and not *Scolelepis*. From our analyses we cannot confirm if the diet preference in *H. hectica* can be explained by the different putative teretoxins expressed in its venom gland. However, contrary to other terebrids examined, *H. hectica* are found in the intertidal zone in the surf. Their restricted habit could be why they are the only ones that feed on *Neanthes* and not *Scolelepis* which are also found in the surf. *H. hectica* results highlight how important it is to examine teretoxins and diet within the context of a single individual. The tremendous amount of variation in terebrid foregut anatomy can possibly be explained by the niche variation hypothesis. The niche variation hypothesis indicates “[among-species] populations with wider niches are more variable than populations with narrower niches” [31]. The terebrids, which are globally distributed and found at bathymetric sea levels ranging from intertidal to up to the pelagic zones, have broadened their niches and their diet so that they can overlap in geographical location and possibly even niche, but still do not have to over-compete for food [26]. Notably, we recently determined environmental pressures may have more of an influence on terebrid diversification than the presence of venom [17].

Overall, we determined that terebrid diet breadth can be correlated with the presence or absence of the venom gland. Specifically, those terebrid species used in this study with a venom gland showed a more restricted diet than those without it. On the other hand, our data suggest that other factors, such as terebrid species classification, shell size, collecting depth, biogeography, and venom complexity, do not significantly influence diet diversity. Additionally, if we consider also the qualitative data we collected, these suggest that, apart from *H. hectica*, terebrids overlap in the prey genera that they consume. Our findings highlight several possibilities about terebrid venom evolution, such as: (1) terebrid species that lack a venom gland may still produce venom via a different tissue, such as salivary glands, allowing them to capture and consume the same genera of annelid prey. It has been reported that the salivary glands of members of the family Conidae (e.g., *Conus pulicarius*), phylogenetically close to Terebridae, can produce venom toxins, as well as those of more distant taxa such as leeches, ticks, and others [32,33,34,35,36,37]. Indeed, several terebrid species used in our study lack a venom gland but have salivary glands. (2) Terebrids without any components of the venom apparatus (venom gland, proboscis, radular sac) may either release venom directly into the water, as has been document from cone snails, or may forego using venom to subdue their prey. If this is true, it could explain why the traditional venom gland has been lost on eight occasions in the Terebridae [19]. Taken together, our findings and those of Phoung and colleagues suggest the current method of classifying cono- or tere-toxins is lacking in details and proposes that venom composition in conoidean snails should be redefined to more accurately reflect the complexity. A holistic model in which we examine, both number of toxins, cysteine framework, gene superfamilies, and prey composition may provide more valid analyses of venom arsenal classifications. Finally, this work illuminates the importance of examining neglected venom organisms in constructing robust theories about venom evolution and species diversification.

## 4. Materials and Methods

### 4.1. DNA Extraction and Amplification for Next Generation Sequencing

Seventy-one terebrid species (*n* = 71) collected in Kavieng, Papua New Guinea (*n* = 60), Tampa, Florida (*n* = 4), and Fujairah, United Arab Emirates (*n* = 7) were chosen from five phylogenetic clades to represent the diverse foreguts found in the Terebridae family. DNA was extracted using the following phenol chloroform extraction protocol. Dissected guts were lysed overnight in lysis buffer and proteinase K at 56 °C. After centrifuging the samples post incubation, 200 µL phenol was added. Samples were centrifuged at 13,000 rpm and the supernatant was added to 100 µL phenol and 100 µL chloroform. Following centrifugation, the supernatant was added to 200 µL chloroform and centrifuged. The supernatant was added to 20 µL sodium acetate (3M, pH 5.2) and 400 µL 95% ethanol. After 2 h resting at −20 °C, samples were centrifuged and the resulting pellet was washed in 500 µL 70% ethanol. The pellet was collected and suspended in 50 µL Tris EDTA buffer (pH 8) and stored at −20 °C. DNA was quantified usinga Qubit fluorometer (Invitrogen, Carlsbad, CA, USA).

### 4.2. PCR Primers and Amplification

We established a set of PCR primers and a PCR protocol that optimized the amplification of the 16S rDNA gene in marine worms. The PCR primers should ideally find a balance between the short, degraded prey DNA found in the guts and a viable sequence length necessary for identifying the prey. We aimed to optimize primers that would amplify a wide range of marine worm DNA, the known prey of terebrids, without amplifying the terebrid DNA. For this study, we amplified a ~350 bp fragment of the 16S rDNA gene using a pair of primers designed to amplify worms DNA and to exclude that from the mollusc: 16SANNF3 (GTATCCTGACCGTGCWAAGGTAGC) designed by 37, and 16Spr1_NP (CCTAAGCCAACATCGAGGTGC) which was modified from the primer 16Spr1 by 37 to not amplify turrid 16S rDNA as well (N. Puillandre, pers. com.). The efficiency of primers was tested by amplifying a mix of DNA containing terebrid and marine worm DNA in the ratios 100:1 and 1000:1, respectively. PCR products were confirmed on a 1.3% agarose gel stained with GelRed.

PCR was carried out in 25 µL volumes with a final concentration of 1× of the supplied PCR buffer, Platinum Hot Start PCR Master Mix 2X (Invitrogen, Carlsbad, CA, USA) and 0.2 µM of each forward and reverse primer. For each reaction, 1 µL of the extracted DNA was added. The thermocycler protocol was 94 °C, followed by 40 cycles of 94 °C for 20 s, 60 °C for 20 s, and 72 °C for a minute. The extension period lasted for 5 min at 72 °C and the PCR products were cooled at 17 °C and stored at −20 °C following PCR product confirmation. Confirmation of PCR products was based on the presence of a band in a 1.3% agarose gel stained with GelRed.

### 4.3. Library Preparation and Sequencing

Libraries were prepared using the Nextera XT DNA Sample Preparation Kit (Illumina, San Diego, CA, USA) according to the manufacturer’s protocol [38], unless otherwise stated. Following Illumina’s (Illumina, San Diego, CA, USA) technical note for cluster optimization and the resultant quantity of data each library was normalized for sequencing to 8 pM according to the manufacturer’s protocol. The libraries were quantified using both a Qubit Fluorometer and a 2100 Bioanalyzer (Agilent Technologies, Santa Clara, CA, USA) and equimolar amounts of DNA were pooled from each sample. Three pools were created with approximately 30 samples per pool, to increase the amount of reads per sample. The three pooled samples were each sequenced at the Hunter College/CTBR Bioinformatics sequencing facility, on an Illumina MiSeq, using the Nano Kit V2 chemistry with 500 cycles and a PhiX spike in of 5%, resulting in 300 bp paired end reads (Illumina, San Diego, CA, USA).

### 4.4. Bioinformatics Pipeline

Samples were demultiplexed using CASAVA (Illumina, San Diego, CA, USA) allowing no mismatch per barcode. Remaining adapters were trimmed from the reads with Trimmomatic using the provided Truseq adapter sequences [39]. Paired reads were merged using FLASH [40] with a minimum overlap of 250. FastX Toolkit [41] was used to quality filter reads with a minimum of 60% of bases ≥Q30 and a minimum length of 150 bp and was used to trim PCR primer sequences. Trimmed sequences were then clustered at 97% sequence identity using the cd-hit-est algorithm available from CD-HIT [42]. All resulting sequence clusters were screened for chimeras using cd-hit-dup in CD-HIT [42].

### 4.5. Reads Identification

Obtained sequences were BLASTed against nr database using an e-value cutoff of 1 × 10^−5^ to discard eventual predator sequences and contaminations. For each sequenced specimen, retained prey sequences were added to a database of ~400 16S rDNA sequences retrieved from NCBI coming from a range of polychaete and sipuncula families. Sequences were aligned using MAFFT [43] and maximum likelihood trees were produced using RAxML [44,45] on the CIPRES portal [46]. Due to the incompleteness of the 16S rDNA polychaetes NCBI database at the species level, we considered it more reliable to identify prey sequences at genus level. All sequences included in each phylogenetic clade were manually inspected and a representative one was chosen and identified with the nearest genus in the tree. Because of the inhomogeneity of our sequencings, we only considered our data to represent the presence or absence of annelid genera. Gut content raw reads (PRJNA662924) and 16S rDNA isolated prey sequences (MW007415–MW007668) are available at GenBank (Supplemental Table S2).

### 4.6. Diet Data Analysis

For each prey genus detected, we reported the number of predator specimens and species (absolute number and percentage frequency) and the sampling localities, the presence/absence of the venom apparatus, and the phylogenetic clade of the predators.

For estimating the prey width, the Shannon’s diversity index (H’) was calculated with Past3 [47] at the specimen and species level using prey presence/absence data. The Jaccard similarity coefficient, calculated on the same data with Past3 [47], was used to graphically compare predator diets and the results were displayed as heat matrices produced with R [48]. Shannon’s diversity values were then categorized by species, presence/absence of venom apparatus, phylogenetic clade, collecting macro area (Florida, UAE, and Papua New Guinea), and shell length, and obtained distributions were compared using the Kruskal Wallis non-parametric test calculated with Past3 [47]. A randomization test was also conducted to further corroborate the results. Data from all specimens were randomized by column 100 times, the differences in Shannon indices were recalculated, and tested for significance, using R [48]. Using our in-house Terebridae database, we recovered the maximum, minimum, and average collection depth of each species as well as the depth range, and we plotted these data against the species Shannon’s index with Past3 [47].

For investigating correlations between terebrid venom complexity and diet diversity, the transcriptomes from six terebrid specimens (*Hastula hectica* KVG_110, *Hastula matheroniana* KVG_193, *Terebra subulata* KVG_46, *Terebra guttata* KVG_260, *Terebra argus* KVG_265, and *Terebra straminea* KVG_229) were compared with the results of their gut content eDNA. In particular, the number of total putative terebrid toxins and the number of cysteine frameworks obtained following the bioinformatic pipeline from [49] were plotted against the respective average Shannon’s diversity index, using Past3 [47].

## Data Availability

Publicly available datasets were analyzed in this study. This data can be found here: GenBank https://www.ncbi.nlm.nih.gov/genbank/ (accessed on 29 January 2021), with accession number: PRJNA662924, MW013507, MW007415–MW007668.

## Supplementary Materials

The following are available online at https://www.mdpi.com/2072-6651/13/2/108/s1. Figure S1: Correlation of terebrid diet and phylogenetic clade and shell length; Table S1: Terebridae vouchers number, foregut type, venom apparatus type, phylogenetic clade, shell length, collection locality, and prey genera found in each specimen; Table S2: Prey sequences number, ID, and GenBank accession number. ID and voucher number of the terebrid from which it was isolated.
