# Supplementary material for: Diet Diversity in Carnivorous Terebrid Snails Is Tied to the Presence and Absence of a Venom Gland
Source: Toxins (Basel). 2021 Feb 2;13(2):108. doi: 10.3390/toxins13020108 (PMC7912948; doi:10.3390/toxins13020108)
Supplement: Supplementary file 1 [file toxins-13-00108-s001.pdf]

Supplementary Materials: Diet Diversity in the Carnivorous Terebrid Snails is Tied to the Presence and Absence of a Venom Gland

Juliette Gorson, Giulia Fassio, Emily S. Lau and Mandë Holford

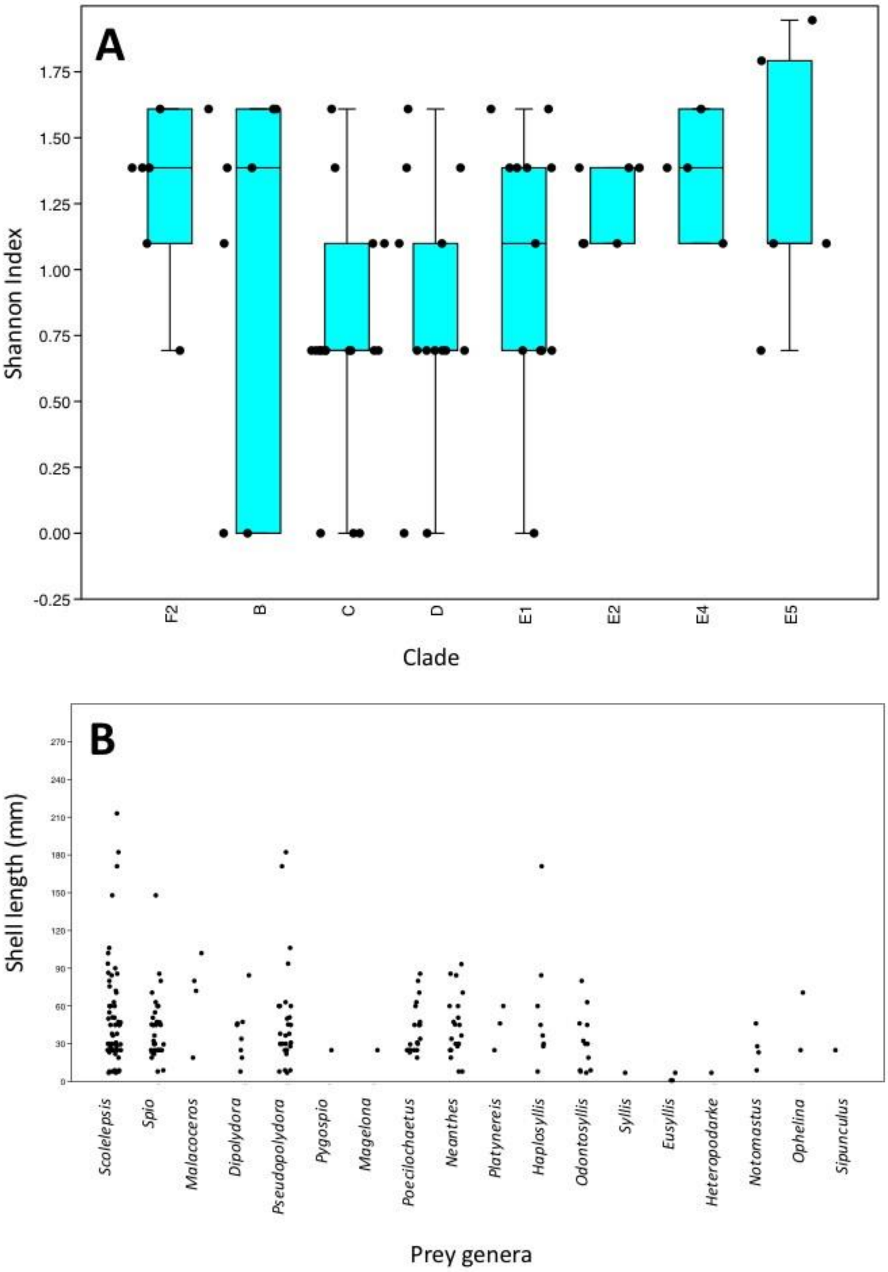

Figure S1. Correlation of terebrid diet and phylogenetic clade (A) and shell length (B). There is no statistical correlation between diet breadth and phylogenetic clade, but we do see that clades C, D, and E2 have the lowest gut diversity, while clade B has the highest gut diversity. Similarly, we do not see a statistical correlation between shell length and genera of prey consumed, but our data is biased because the majority of samples being analyzed are <90 mm.

Table S1. Terebridae vouchers number, foregut type, venom apparatus type, phylogenetic clade, shell length, collection locality, and prey genera found in each specimen (0=absence, 1=presence.).

								**Annelida**	**Annelida**	**Annelida**	**Annelida**	**Annelida**	**Annelida**	**Annelida**	**Annelida**	**Annelida**	**Annelida**	**Annelida**	**Annelida**	**Annelida**	**Annelida**	**Annelida**	**Annelida**	**Annelida**	**Sipuncula**
								Sedentaria	Sedentaria	Sedentaria	Sedentaria	Sedentaria	Sedentaria	Sedentaria	Sedentaria	Errantia	Errantia	Errantia	Errantia	Errantia	Errantia	Errantia	Sedentaria	Sedentaria	
								Spioniformia	Spioniformia	Spioniformia	Spioniformia	Spioniformia	Spioniformia	Spioniformia	Spioniformia	Nereidiformia	Nereidiformia	Nereidiformia	Nereidiformia	Nereidiformia	Nereidiformia	Nereidiformia			
								Spionidae	Spionidae	Spionidae	Spionidae	Spionidae	Spionidae	Magelonidae	Poecilochaetidae	Nereididae	Nereididae	Syllidae	Syllidae	Syllidae	Syllidae	Hesionidae	Opheliidae/Capitellidae	Travisiidae/Opheliidae	Sipunculidae
Voucher #	Species	Foregt type	Venom apparatus type	Phylogeny Clade	Shell length (mm)	Collection Locality	Total *n* of prey types	*Scolelepis*	*Spio*	*Malacoceros*	*Polydora/Dipolydora*	*Pseudopolydora*	*Pygospio*	*Magelona*	*Poecilochaetus*	*Neanthes*	*Platynereis*	*Haplosyllis*	*Odontosyllis*	*Syllis*	*Eusyllis*	*Heteropodarke*	*Ophelia/Notomastus*	*Travisia/Ophelina*	*Sipunculus*
KVG_236	*Hastula acumen*	2	1	D	25	Kavieng, Papua New Guinea	4	1	1	0	1	0	0	0	0	0	1	0	0	0	0	0	0	0	0
KVG_243	*Hastula acumen*	2	1	D	28	Kavieng, Papua New Guinea	2	1	0	0	0	1	0	0	0	0	0	0	0	0	0	0	0	0	0
KVG_201	*Hastula acumen*	2	1	D	30	Kavieng, Papua New	3	1	0	0	0	1	0	0	0	0	0	0	1	0	0	0	0	0	0

						Guinea																			
KVG_203	*Hastula acumen*	2	1	D	30	Kavieng, Papua New Guinea	4	1	1	0	0	1	0	0	0	0	0	0	1	0	0	0	0	0	0
KVG_242	*Hastula acumen*	2	1	D	75.639	Kavieng, Papua New Guinea	1	1	0	0	0	0	0	0	0	0	0	0	0	0	0	0	0	0	0
KVG_198	*Hastula acumen*	2	1	D	30	Kavieng, Papua New Guinea	3	1	0	0	0	1	0	0	0	1	0	0	0	0	0	0	0	0	0
KVG_103	*Myurella affinis*	3	0	E1	31	Kavieng, Papua New Guinea	3	1	0	0	0	1	0	0	1	0	0	0	0	0	0	0	0	0	0
KVG_215	*Myurella affinis*	3	0	E1	38	Kavieng, Papua New Guinea	2	1	0	0	0	1	0	0	0	0	0	0	0	0	0	0	0	0	0
KVG_214	*Myurella affinis*	3	0	E1	46.209	Kavieng, Papua New Guinea	4	0	0	0	1	0	0	0	0	0	1	0	1	0	0	0	1	0	0
KVG_90	*Myurella affinis*	3	0	E1	93.688	Kavieng, Papua New Guinea	2	1	0	0	0	1	0	0	0	0	0	0	0	0	0	0	0	0	0
KVG_97	*Myurella affinis*	3	0	E1	28.071	Kavieng, Papua New Guinea	4	1	0	0	0	0	0	0	0	1	0	1	0	0	0	0	1	0	0
KVG_234	*Hastula albula*	2	1	D	25	Kavieng, Papua New Guinea	2	0	0	0	0	1	0	0	0	0	0	0	0	0	0	0	0	1	0
KVG_53	*Myurella amoena*	3	0	E1	23.21	Kavieng, Papua New Guinea	4	1	1	0	0	0	0	0	1	0	0	0	0	0	0	0	1	0	0
KVG_159	*Myurella amoena*	3	0	E1	36.6	Kavieng,	5	1	1	0	0	1	0	0	0	1	0	1	0	0	0	0	0	0	0

						Papua New Guinea																			
KVG_2	*Myurella amoena*	3	0	E1	70.676	Kavieng, Papua New Guinea	5	1	1	0	0	0	0	0	1	1	0	0	0	0	0	0	0	1	0
KVG_247	*Oxymeris areolata*	1	0	B	86.424	Kavieng, Papua New Guinea	1	1	0	0	0	0	0	0	0	0	0	0	0	0	0	0	0	0	0
KVG_41	*Oxymeris areolata*	1	0	B	213.001	Kavieng, Papua New Guinea	1	1	0	0	0	0	0	0	0	0	0	0	0	0	0	0	0	0	0
KVG_190	*Oxymeris areolata*	1	0	B	34	Kavieng, Papua New Guinea	3	0	0	0	1	0	0	0	1	1	0	0	0	0	0	0	0	0	0
KVG_265	*Terebra argus*	2	1	C	51	Kavieng, Papua New Guinea	2	1	0	0	0	1	0	0	0	0	0	0	0	0	0	0	0	0	0
KVG_129	*Terebra argus*	2	1	C	147.93	Kavieng, Papua New Guinea	2	1	1	0	0	0	0	0	0	0	0	0	0	0	0	0	0	0	0
KVG_XX	*Terebra argus*	2	1	C	?	Kavieng, Papua New Guinea	4	1	1	0	0	1	0	0	1	0	0	0	0	0	0	0	0	0	0
KVG_84	*Terebra argus*	2	1	C	47.376	Kavieng, Papua New Guinea	5	1	1	0	1	0	0	0	1	1	0	0	0	0	0	0	0	0	0
Tamp_17	*Neoterebra dislo-cata*	3	0	E4	45	Tampa, Florida, USA	3	1	1	0	0	0	0	0	1	0	0	0	0	0	0	0	0	0	0
Tamp_18	*Neoterebra dislo-cata*	3	0	E4	45	Tampa, Florida, USA	4	1	1	0	1	0	0	0	0	0	0	1	0	0	0	0	0	0	0
Tamp_63	*Neoterebra dislo-cata*	3	0	E4	45	Tampa, Florida, USA	4	1	1	0	0	0	0	0	1	0	0	0	1	0	0	0	0	0	0
Tamp_50	*Neoterebra dislo-cata*	3	0	E4	45	Tampa, Florida,	5	1	1	0	0	1	0	0	1	1	0	0	0	0	0	0	0	0	0

						USA																		
KVG_113	*Oxymeris felina*	1	0	B	63.051	Kavieng, Papua New Guinea	5	1	1	0	0	1	0	0	1	0	0	0	1	0	0	0	0	0	0
KVG_211	*Oxymeris felina*	1	0	B	80	Kavieng, Papua New Guinea	5	1	1	1	0	0	0	0	1	0	0	0	1	0	0	0	0	0	0
KVG_112	*Oxymeris felina*	1	0	B	29.587	Kavieng, Papua New Guinea	4	1	1	0	0	0	0	0	1	1	0	0	0	0	0	0	0	0	0
KVG_114	*Oxymeris felina*	1	0	B	85.667	Kavieng, Papua New Guinea	4	1	1	0	0	0	0	0	1	1	0	0	0	0	0	0	0	0	0
KVG_26	*Terebra guttata*	2	1	C	25	Kavieng, Papua New Guinea	2	1	0	0	0	0	1	0	0	0	0	0	0	0	0	0	0	0	0
KVG_108	*Terebra guttata*	2	1	C	32.315	Kavieng, Papua New Guinea	2	0	1	0	0	0	0	0	0	0	0	0	1	0	0	0	0	0	0
KVG_260	*Terebra guttata*	2	1	C	55	Kavieng, Papua New Guinea	2	1	1	0	0	0	0	0	0	0	0	0	0	0	0	0	0	0	0
KVG_256	*Terebra guttata*	2	1	C	90	Kavieng, Papua New Guinea	1	1	0	0	0	0	0	0	0	0	0	0	0	0	0	0	0	0	0
KVG_66	*Terebra guttata*	2	1	C	171.048	Kavieng, Papua New Guinea	3	1	0	0	0	1	0	0	0	0	0	1	0	0	0	0	0	0	0
KVG_249	*Hastula hectica*	2	1	D	60	Kavieng, Papua New Guinea	5	1	1	0	0	1	0	0	0	0	1	1	0	0	0	0	0	0	0
KVG_253	*Hastula hectica*	2	1	D	60	Kavieng, Papua New Guinea	2	1	0	0	0	1	0	0	0	0	0	0	0	0	0	0	0	0	0
KVG_58	*Hastula hectica*	2	1	D	60	Kavieng, Papua New Guinea	1	1	0	0	0	0	0	0	0	0	0	0	0	0	0	0	0	0	0
KVG_110	*Hastula hectica*	2	1	D	60	Kavieng,	2	0	0	0	0	1	0	0	0	1	0	0	0	0	0	0	0	0	0

						Papua New Guinea																			
KVG_54	*Hastula hectica*	2	1	D	60	Kavieng, Papua New Guinea	2	1	0	0	0	0	0	0	0	1	0	0	0	0	0	0	0	0	0
KVG_193	*Hastula matheroniana*	2	1	D	30	Kavieng, Papua New Guinea	2	1	1	0	0	0	0	0	0	0	0	0	0	0	0	0	0	0	0
FJ_10	*Partecosta nassoides*	1	0	F2	7	Fujairah, United Arab Emirates	5	1	0	0	0	0	0	0	0	0	0	0	1	1	1	1	0	0	0
FJ_15	*Partecosta nassoides*	1	0	F2	7	Fujairah, United Arab Emirates	2	1	0	0	0	1	0	0	0	0	0	0	0	0	0	0	0	0	0
FJ_12	*Partecosta nassoides*	1	0	F2	8	Fujairah, United Arab Emirates	4	1	1	0	1	0	0	0	0	0	0	0	1	0	0	0	0	0	0
FJ_13	*Partecosta nassoides*	1	0	F2	9	Fujairah, United Arab Emirates	4	1	0	0	0	1	0	0	0	0	0	0	1	0	0	0	1	0	0
FJ_14	*Partecosta nassoides*	1	0	F2	9	Fujairah, United Arab Emirates	5	1	1	0	0	1	0	0	0	0	0	0	1	0	1	0	0	0	0
FJ_11	*Partecosta nassoides*	1	0	F2	8	Fujairah, United Arab Emirates	4	1	0	0	0	1	0	0	0	1	0	0	0	0	1	0	0	0	0
FJ_16	*Partecosta nassoides*	1	0	F2	8	Fujairah, United Arab Emirates	3	1	0	0	0	0	0	0	0	1	0	1	0	0	0	0	0	0	0
KVG_127	*Myurella nebulosa*	3	0	E1	47	Kavieng, Papua New	2	1	1	0	0	0	0	0	0	0	0	0	0	0	0	0	0	0	0

						Guinea																			
KVG_50	*Myurella nebulosa*	3	0	E1	50	Kavieng, Papua New Guinea	2	1	0	0	0	1	0	0	0	0	0	0	0	0	0	0	0	0	0
KVG_48	*Myurella nebulosa*	3	0	E1	84.381	Kavieng, Papua New Guinea	4	1	0	0	1	0	0	0	0	1	0	1	0	0	0	0	0	0	0
KVG_176	*Myurella nebulosa*	3	0	E1	93.26	Kavieng, Papua New Guinea	1	0	0	0	0	0	0	0	0	1	0	0	0	0	0	0	0	0	0
KVG_136	*Punctoterebra roseata*	3	0	E2	25	Kavieng, Papua New Guinea	3	1	0	0	0	0	0	1	1	0	0	0	0	0	0	0	0	0	0
KVG_137	*Punctoterebra roseata*	3	0	E2	25	Kavieng, Papua New Guinea	4	1	1	0	0	1	0	0	1	0	0	0	0	0	0	0	0	0	0
KVG_140	*Punctoterebra roseata*	3	0	E2	25	Kavieng, Papua New Guinea	3	1	1	0	0	0	0	0	1	0	0	0	0	0	0	0	0	0	0
KVG_138	*Punctoterebra roseata*	3	0	E2	25	Kavieng, Papua New Guinea	4	1	1	0	0	0	0	0	1	1	0	0	0	0	0	0	0	0	0
KVG_142	*Punctoterebra roseata*	3	0	E2	25	Kavieng, Papua New Guinea	4	1	1	0	0	0	0	0	1	1	0	0	0	0	0	0	0	0	0
KVG_139	*Punctoterebra roseata*	3	0	E2	25	Kavieng, Papua New Guinea	3	1	1	0	0	0	0	0	0	0	0	0	0	0	0	0	0	0	1
KVG_229	*Terebra straminea*	2	1	C	106.166	Kavieng, Papua New Guinea	2	1	0	0	0	1	0	0	0	0	0	0	0	0	0	0	0	0	0
KVG_146	*Terebra subulata*	2	1	C	38	Kavieng, Papua New Guinea	2	1	0	0	0	1	0	0	0	0	0	0	0	0	0	0	0	0	0
KVG_145	*Terebra subulata*	2	1	C	46	Kavieng, Papua New Guinea	1	1	0	0	0	0	0	0	0	0	0	0	0	0	0	0	0	0	0
KVG_268	*Terebra subulata*	2	1	C	72.053	Kavieng,	2	1	0	1	0	0	0	0	0	0	0	0	0	0	0	0	0	0	0

						Papua New Guinea																			
KVG_144	*Terebra subulata*	2	1	C	102	Kavieng, Papua New Guinea	2	1	0	1	0	0	0	0	0	0	0	0	0	0	0	0	0	0	0
KVG_47	*Terebra subulata*	2	1	C	182.209	Kavieng, Papua New Guinea	2	1	0	0	0	1	0	0	0	0	0	0	0	0	0	0	0	0	0
KVG_46	*Terebra subulata*	2	1	C	?	Kavieng, Papua New Guinea	1	1	0	0	0	0	0	0	0	0	0	0	0	0	0	0	0	0	0
KVG_92	*Terebra subulata*	2	1	C	50.71	Kavieng, Papua New Guinea	3	1	1	0	0	0	0	0	0	1	0	0	0	0	0	0	0	0	0
KVG_156	*Myurellopsis undulata*	3	0	E5	22	Kavieng, Papua New Guinea	3	1	1	0	0	1	0	0	0	0	0	0	0	0	0	0	0	0	0
KVG_60	*Myurellopsis undulata*	3	0	E5	60	Kavieng, Papua New Guinea	2	0	1	0	0	0	0	0	1	0	0	0	0	0	0	0	0	0	0
KVG_105	*Myurellopsis undulata*	3	0	E5	19	Kavieng, Papua New Guinea	7	1	1	1	1	0	0	0	1	1	0	0	1	0	0	0	0	0	0
KVG_155	*Myurellopsis undulata*	3	0	E5	30	Kavieng, Papua New Guinea	6	1	1	0	0	1	0	0	1	1	0	1	0	0	0	0	0	0	0
KVG_5	*Myurellopsis undulata*	3	0	E5	45.358	Kavieng, Papua New Guinea	3	0	1	0	0	1	0	0	0	1	0	0	0	0	0	0	0	0	0

Table S2. Prey sequences number, ID, and GenBank accession number. ID and voucher number of the terebrid from which it was isolated.

Prey Sequence #	Prey Sequence ID	Terebrid ID	Terebrid Voucher #	GenBank Accession Number
15535_11242	*Eusyllis*	*Partecosta nassoides*	FJ_10	MW007415
27741_18278	*Heteropodarke*	*Partecosta nassoides*	FJ_10	MW007416
9150_27499	*Odontosyllis*	*Partecosta nassoides*	FJ_10	MW007417
13263_27555	*Odontosyllis*	*Partecosta nassoides*	FJ_10	MW007418
23034_23948	*Scolelepis*	*Partecosta nassoides*	FJ_10	MW007419
7820_24829	*Syllis*	*Partecosta nassoides*	FJ_10	MW007420
14802_6000	*Eusyllis*	*Partecosta nassoides*	FJ_11	MW007421
7824_12340	*Neanthes*	*Partecosta nassoides*	FJ_11	MW007422
24518_17679	*Pseudopolydora*	*Partecosta nassoides*	FJ_11	MW007423
20557_11191	*Scolelepis*	*Partecosta nassoides*	FJ_11	MW007424
17551_11858	*Scolelepis*	*Partecosta nassoides*	FJ_11	MW007425
14778_2401	*Odontosyllis*	*Partecosta nassoides*	FJ_12	MW007426
18320_11226	*Polydora_Dipolydora*	*Partecosta nassoides*	FJ_12	MW007427
26839_22477	*Scolelepis*	*Partecosta nassoides*	FJ_12	MW007428
27889_14521	*Scolelepis*	*Partecosta nassoides*	FJ_12	MW007429
22811_11823	*Scolelepis*	*Partecosta nassoides*	FJ_12	MW007430
18617_17154	*Spio*	*Partecosta nassoides*	FJ_12	MW007431
6985_6145	*Odontosyllis*	*Partecosta nassoides*	FJ_13	MW007432
12115_17716	*Odontosyllis*	*Partecosta nassoides*	FJ_13	MW007433
24746_14609	*Ophelia_Notomastus*	*Partecosta nassoides*	FJ_13	MW007434
18185_8609	*Pseudopolydora*	*Partecosta nassoides*	FJ_13	MW007435
20015_3213	*Scolelepis*	*Partecosta nassoides*	FJ_13	MW007436
16398_11181	*Odontosyllis*	*Partecosta nassoides*	FJ_14	MW007437
11877_26991	*Eusyllis*	*Partecosta nassoides*	FJ_14	MW007438
12674_14511	*Pseudopolydora*	*Partecosta nassoides*	FJ_14	MW007439
16144_16431	*Scolelepis*	*Partecosta nassoides*	FJ_14	MW007440
20107_18305	*Spio*	*Partecosta nassoides*	FJ_14	MW007441
14007_27289	*Spio*	*Partecosta nassoides*	FJ_14	MW007442
15317_22380	*Pseudopolydora*	*Partecosta nassoides*	FJ_15	MW007443
5803_18923	*Scolelepis*	*Partecosta nassoides*	FJ_15	MW007444
25615_9879	*Scolelepis*	*Partecosta nassoides*	FJ_15	MW007445
18264_135	*Haplosyllis*	*Partecosta nassoides*	FJ_16	MW007446
18848_117	*Neanthes*	*Partecosta nassoides*	FJ_16	MW007447
28680_20547	*Scolelepis*	*Partecosta nassoides*	FJ_16	MW007448
19594_17270	*Poecilochaetus*	*Myurella affinis*	KVG_103	MW007449
23950_23805	*Pseudopolydora*	*Myurella affinis*	KVG_103	MW007450
9593_5822	*Scolelepis*	*Myurella affinis*	KVG_103	MW007451
14898_25186	*Scolelepis*	*Myurella affinis*	KVG_103	MW007452
19111_2419	*Malacoceros*	*Myurellopsis undulata*	KVG_105	MW007453
18144_16156	*Neanthes*	*Myurellopsis undulata*	KVG_105	MW007454
13901_18332	*Odontosyllis*	*Myurellopsis undulata*	KVG_105	MW007455
2470_12396	*Poecilochaetus*	*Myurellopsis undulata*	KVG_105	MW007456
23663_18659	*Polydora_Dipolydora*	*Myurellopsis undulata*	KVG_105	MW007457
15354_6474	*Scolelepis*	*Myurellopsis undulata*	KVG_105	MW007458
17650_19004	*Spio*	*Myurellopsis undulata*	KVG_105	MW007459
8684_18404	*Spio*	*Myurellopsis undulata*	KVG_105	MW007460

23854_18844	*Odontosyllis*	*Terebra guttata*	KVG_108	MW007461
16882_8371	*Spio*	*Terebra guttata*	KVG_108	MW007462
26156_6985	*Neanthes*	*Hastula hectica*	KVG_110	MW007463
20691_12826	*Pseudopolydora*	*Hastula hectica*	KVG_110	MW007464
22470_17554	*Neanthes*	*Oxymeris felina*	KVG_112	MW007465
28004_19868	*Poecilochaetus*	*Oxymeris felina*	KVG_112	MW007466
14055_2817	*Scolelepis*	*Oxymeris felina*	KVG_112	MW007467
1865_14120	*Spio*	*Oxymeris felina*	KVG_112	MW007468
23689_26364	*Scolelepis*	*Oxymeris felina*	KVG_113	MW007469
11753_22594	*Odontosyllis*	*Oxymeris felina*	KVG_113	MW007470
7142_21217	*Odontosyllis*	*Oxymeris felina*	KVG_113	MW007471
8032_7103	*Poecilochaetus*	*Oxymeris felina*	KVG_113	MW007472
26929_16407	*Pseudopolydora*	*Oxymeris felina*	KVG_113	MW007473
11416_3306	*Spio*	*Oxymeris felina*	KVG_113	MW007474
16407_4906	*Neanthes*	*Oxymeris felina*	KVG_114	MW007475
16630_21377	*Poecilochaetus*	*Oxymeris felina*	KVG_114	MW007476
7221_4774	*Scolelepis*	*Oxymeris felina*	KVG_114	MW007477
26308_16440	*Spio*	*Oxymeris felina*	KVG_114	MW007478
22628_22760	*Scolelepis*	*Myurella nebulosa*	KVG_127	MW007479
27330_14803	*Scolelepis*	*Myurella nebulosa*	KVG_127	MW007480
5691_23032	*Spio*	*Myurella nebulosa*	KVG_127	MW007481
9176_25384	*Scolelepis*	*Terebra argus*	KVG_129	MW007482
18419_28469	*Spio*	*Terebra argus*	KVG_129	MW007483
26460_20014	*Magelona*	*Punctoterebra roseata*	KVG_136	MW007484
19939_25342	*Poecilochaetus*	*Punctoterebra roseata*	KVG_136	MW007485
8247_8407	*Scolelepis*	*Punctoterebra roseata*	KVG_136	MW007486
6542_5183	*Scolelepis*	*Punctoterebra roseata*	KVG_137	MW007487
12255_4418	*Poecilochaetus*	*Punctoterebra roseata*	KVG_137	MW007488
8270_18053	*Pseudopolydora*	*Punctoterebra roseata*	KVG_137	MW007489
28847_10744	*Scolelepis*	*Punctoterebra roseata*	KVG_137	MW007490
4916_16212	*Scolelepis*	*Punctoterebra roseata*	KVG_137	MW007491
13894_25373	*Spio*	*Punctoterebra roseata*	KVG_137	MW007492
16237_20640	*Spio*	*Punctoterebra roseata*	KVG_137	MW007493
5634_9564	*Neanthes*	*Punctoterebra roseata*	KVG_138	MW007494
23363_20092	*Poecilochaetus*	*Punctoterebra roseata*	KVG_138	MW007495
18559_28088	*Scolelepis*	*Punctoterebra roseata*	KVG_138	MW007496
22221_6388	*Scolelepis*	*Punctoterebra roseata*	KVG_138	MW007497
23351_20524	*Scolelepis*	*Punctoterebra roseata*	KVG_138	MW007498
22818_7965	*Spio*	*Punctoterebra roseata*	KVG_138	MW007499
26407_18192	*Scolelepis*	*Punctoterebra roseata*	KVG_139	MW007500
11040_16772	*Sipunculus*	*Punctoterebra roseata*	KVG_139	MW007501
22876_26268	*Spio*	*Punctoterebra roseata*	KVG_139	MW007502
13614_12805	*Poecilochaetus*	*Punctoterebra roseata*	KVG_140	MW007503
4051_13103	*Scolelepis*	*Punctoterebra roseata*	KVG_140	MW007504
7509_4516	*Scolelepis*	*Punctoterebra roseata*	KVG_140	MW007505
4449_9589	*Spio*	*Punctoterebra roseata*	KVG_140	MW007506
24206_16262	*Neanthes*	*Punctoterebra roseata*	KVG_142	MW007507
27522_17392	*Poecilochaetus*	*Punctoterebra roseata*	KVG_142	MW007508
9030_16446	*Scolelepis*	*Punctoterebra roseata*	KVG_142	MW007509
15139_9023	*Spio*	*Punctoterebra roseata*	KVG_142	MW007510
7293_4536	*Spio*	*Punctoterebra roseata*	KVG_142	MW007511

17046_9851	*Spio*	*Punctoterebra roseata*	KVG_142	MW007512
24701_6478	*Malacoceros*	*Terebra subulata*	KVG_144	MW007513
14975_22281	*Scolelepis*	*Terebra subulata*	KVG_144	MW007514
23987_22102	*Scolelepis*	*Terebra subulata*	KVG_144	MW007515
19169_3050	*Scolelepis*	*Terebra subulata*	KVG_144	MW007516
27440_10843	*Scolelepis*	*Terebra subulata*	KVG_145	MW007517
8290_19193	*Pseudopolydora*	*Terebra subulata*	KVG_146	MW007518
12602_26516	*Scolelepis*	*Terebra subulata*	KVG_146	MW007519
12265_17512	*Scolelepis*	*Terebra subulata*	KVG_146	MW007520
21945_21465	*Haplosyllis*	*Myurellopsis undulata*	KVG_155	MW007521
13998_13312	*Neanthes*	*Myurellopsis undulata*	KVG_155	MW007522
28428_18875	*Poecilochaetus*	*Myurellopsis undulata*	KVG_155	MW007523
28203_10583	*Pseudopolydora*	*Myurellopsis undulata*	KVG_155	MW007524
5897_16222	*Scolelepis*	*Myurellopsis undulata*	KVG_155	MW007525
26672_13907	*Scolelepis*	*Myurellopsis undulata*	KVG_155	MW007526
11820_5426	*Spio*	*Myurellopsis undulata*	KVG_155	MW007527
5410_19212	*Pseudopolydora*	*Myurellopsis undulata*	KVG_156	MW007528
23231_5405	*Scolelepis*	*Myurellopsis undulata*	KVG_156	MW007529
23530_17809	*Spio*	*Myurellopsis undulata*	KVG_156	MW007530
14858_26879	*Spio*	*Myurellopsis undulata*	KVG_156	MW007531
26146_16593	*Haplosyllis*	*Myurella amoena*	KVG_159	MW007532
21635_16635	*Neanthes*	*Myurella amoena*	KVG_159	MW007533
14466_12124	*Pseudopolydora*	*Myurella amoena*	KVG_159	MW007534
15513_15064	*Scolelepis*	*Myurella amoena*	KVG_159	MW007535
8116_19971	*Spio*	*Myurella amoena*	KVG_159	MW007536
10842_8049	*Spio*	*Myurella amoena*	KVG_159	MW007537
11319_18350	*Neanthes*	*Myurella nebulosa*	KVG_176	MW007538
12601_2768	*Neanthes*	*Oxymeris areolata*	KVG_190	MW007539
5746_13549	*Poecilochaetus*	*Oxymeris areolata*	KVG_190	MW007540
2342_17037	*Polydora_Dipolydora*	*Oxymeris areolata*	KVG_190	MW007541
17797_8733	*Scolelepis*	*Hastula matheroniana*	KVG_193	MW007542
12238_16883	*Spio*	*Hastula matheroniana*	KVG_193	MW007543
18533_15435	*Neanthes*	*Hastula acumen*	KVG_198	MW007544
26751_17717	*Pseudopolydora*	*Hastula acumen*	KVG_198	MW007545
17643_25322	*Scolelepis*	*Hastula acumen*	KVG_198	MW007546
4009_20186	*Scolelepis*	*Hastula acumen*	KVG_198	MW007547
12388_18081	*Neanthes*	*Myurella amoena*	KVG_2	MW007548
9149_19714	*Poecilochaetus*	*Myurella amoena*	KVG_2	MW007549
6139_6634	*Scolelepis*	*Myurella amoena*	KVG_2	MW007550
4247_17779	*Spio*	*Myurella amoena*	KVG_2	MW007551
3393_16130	*Travisia_Ophelina*	*Myurella amoena*	KVG_2	MW007552
14320_6391	*Odontosyllis*	*Hastula acumen*	KVG_201	MW007553
13805_6516	*Pseudopolydora*	*Hastula acumen*	KVG_201	MW007554
10324_5551	*Scolelepis*	*Hastula acumen*	KVG_201	MW007555
19276_17234	*Scolelepis*	*Hastula acumen*	KVG_201	MW007556
25275_6824	*Odontosyllis*	*Hastula acumen*	KVG_203	MW007557
7246_13919	*Odontosyllis*	*Hastula acumen*	KVG_203	MW007558
26670_14670	*Pseudopolydora*	*Hastula acumen*	KVG_203	MW007559
20298_3603	*Scolelepis*	*Hastula acumen*	KVG_203	MW007560
16589_25836	*Spio*	*Hastula acumen*	KVG_203	MW007561
8902_17154	*Malacoceros*	*Oxymeris felina*	KVG_211	MW007562

19603_24900	*Odontosyllis*	*Oxymeris felina*	KVG_211	MW007563
6477_23447	*Poecilochaetus*	*Oxymeris felina*	KVG_211	MW007564
23390_20095	*Scolelepis*	*Oxymeris felina*	KVG_211	MW007565
9089_11444	*Spio*	*Oxymeris felina*	KVG_211	MW007566
8430_6512	*Spio*	*Oxymeris felina*	KVG_211	MW007567
19676_19282	*Odontosyllis*	*Myurella affinis*	KVG_214	MW007568
11262_12156	*Ophelia_Notomastus*	*Myurella affinis*	KVG_214	MW007569
11065_20081	*Platynereis*	*Myurella affinis*	KVG_214	MW007570
21477_22747	*Polydora_Dipolydora*	*Myurella affinis*	KVG_214	MW007571
9817_13756	*Pseudopolydora*	*Myurella affinis*	KVG_215	MW007572
23181_6333	*Scolelepis*	*Myurella affinis*	KVG_215	MW007573
28009_13589	*Scolelepis*	*Terebra straminea*	KVG_229	MW007574
23183_21366	*Pseudopolydora*	*Terebra straminea*	KVG_229	MW007575
18701_3053	*Scolelepis*	*Terebra straminea*	KVG_229	MW007576
14816_11458	*Pseudopolydora*	*Hastula albula*	KVG_234	MW007577
3995_11379	*Travisia_Ophelina*	*Hastula albula*	KVG_234	MW007578
10383_14630	*Platynereis*	*Hastula acumen*	KVG_236	MW007579
17002_14609	*Polydora_Dipolydora*	*Hastula acumen*	KVG_236	MW007580
16408_5047	*Scolelepis*	*Hastula acumen*	KVG_236	MW007581
4846_7976	*Spio*	*Hastula acumen*	KVG_236	MW007582
23010_17896	*Scolelepis*	*Hastula acumen*	KVG_242	MW007583
19307_12483	*Scolelepis*	*Hastula acumen*	KVG_242	MW007584
19176_2181	*Pseudopolydora*	*Hastula acumen*	KVG_243	MW007585
24823_18358	*Scolelepis*	*Hastula acumen*	KVG_243	MW007586
16033_6921	*Scolelepis*	*Oxymeris areolata*	KVG_247	MW007587
16236_23478	*Haplosyllis*	*Hastula hectica*	KVG_249	MW007588
23562_13630	*Platynereis*	*Hastula hectica*	KVG_249	MW007589
4501_14850	*Pseudopolydora*	*Hastula hectica*	KVG_249	MW007590
23099_5976	*Scolelepis*	*Hastula hectica*	KVG_249	MW007591
14318_9216	*Scolelepis*	*Hastula hectica*	KVG_249	MW007592
8508_19939	*Spio*	*Hastula hectica*	KVG_249	MW007593
27874_22269	*Pseudopolydora*	*Hastula hectica*	KVG_253	MW007594
26823_19500	*Scolelepis*	*Hastula hectica*	KVG_253	MW007595
18567_14060	*Scolelepis*	*Terebra guttata*	KVG_256	MW007596
16952_5608	*Scolelepis*	*Terebra guttata*	KVG_256	MW007597
7949_19305	*Pygospio*	*Terebra guttata*	KVG_26	MW007598
3132_17052	*Scolelepis*	*Terebra guttata*	KVG_26	MW007599
9529_10330	*Scolelepis*	*Terebra guttata*	KVG_260	MW007600
23383_20329	*Spio*	*Terebra guttata*	KVG_260	MW007601
15102_7470	*Pseudopolydora*	*Terebra argus*	KVG_265	MW007602
2742_14021	*Scolelepis*	*Terebra argus*	KVG_265	MW007603
29553_15227	*Malacoceros*	*Terebra subulata*	KVG_268	MW007604
11548_8644	*Scolelepis*	*Terebra subulata*	KVG_268	MW007605
12299_9333	*Scolelepis*	*Oxymeris areolata*	KVG_41	MW007606
5817_7142	*Scolelepis*	*Terebra subulata*	KVG_46	MW007607
21614_5002	*Pseudopolydora*	*Terebra subulata*	KVG_47	MW007608
23673_26468	*Scolelepis*	*Terebra subulata*	KVG_47	MW007609
19429_14576	*Haplosyllis*	*Myurella nebulosa*	KVG_48	MW007610
1921_15447	*Neanthes*	*Myurella nebulosa*	KVG_48	MW007611
22445_4691	*Polydora_Dipolydora*	*Myurella nebulosa*	KVG_48	MW007612
7462_26393	*Scolelepis*	*Myurella nebulosa*	KVG_48	MW007613

15140_19854	*Neanthes*	*Myurellopsis undulata*	KVG_5	MW007614
26066_19002	*Pseudopolydora*	*Myurellopsis undulata*	KVG_5	MW007615
27042_18130	*Spio*	*Myurellopsis undulata*	KVG_5	MW007616
15176_13362	*Pseudopolydora*	*Myurella nebulosa*	KVG_50	MW007617
11713_14876	*Scolelepis*	*Myurella nebulosa*	KVG_50	MW007618
13384_19770	*Ophelia_Notomastus*	*Myurella amoena*	KVG_53	MW007619
12303_28041	*Poecilochaetus*	*Myurella amoena*	KVG_53	MW007620
21756_20025	*Scolelepis*	*Myurella amoena*	KVG_53	MW007621
1883_17254	*Scolelepis*	*Myurella amoena*	KVG_53	MW007622
2597_11868	*Spio*	*Myurella amoena*	KVG_53	MW007623
8463_4508	*Neanthes*	*Hastula hectica*	KVG_54	MW007624
27590_19857	*Scolelepis*	*Hastula hectica*	KVG_54	MW007625
18834_10600	*Scolelepis*	*Hastula hectica*	KVG_58	MW007626
21152_26920	*Poecilochaetus*	*Myurellopsis undulata*	KVG_60	MW007627
9682_3840	*Spio*	*Myurellopsis undulata*	KVG_60	MW007628
24583_6310	*Haplosyllis*	*Terebra guttata*	KVG_66	MW007629
22033_16326	*Pseudopolydora*	*Terebra guttata*	KVG_66	MW007630
13402_5805	*Scolelepis*	*Terebra guttata*	KVG_66	MW007631
9793_23573	*Neanthes*	*Terebra argus*	KVG_84	MW007632
6340_16207	*Poecilochaetus*	*Terebra argus*	KVG_84	MW007633
25735_22851	*Polydora_Dipolydora*	*Terebra argus*	KVG_84	MW007634
23404_12340	*Scolelepis*	*Terebra argus*	KVG_84	MW007635
28220_13266	*Spio*	*Terebra argus*	KVG_84	MW007636
11307_21693	*Spio*	*Terebra argus*	KVG_84	MW007637
8732_10972	*Spio*	*Terebra argus*	KVG_84	MW007638
5405_7597	*Pseudopolydora*	*Myurella affinis*	KVG_90	MW007639
11393_28576	*Scolelepis*	*Myurella affinis*	KVG_90	MW007640
24416_25390	*Neanthes*	*Terebra subulata*	KVG_92	MW007641
12583_12537	*Scolelepis*	*Terebra subulata*	KVG_92	MW007642
5302_23341	*Spio*	*Terebra subulata*	KVG_92	MW007643
24684_21551	*Haplosyllis*	*Myurella affinis*	KVG_97	MW007644
26680_21334	*Neanthes*	*Myurella affinis*	KVG_97	MW007645
14657_10199	*Ophelia_Notomastus*	*Myurella affinis*	KVG_97	MW007646
15757_27301	*Scolelepis*	*Myurella affinis*	KVG_97	MW007647
15250_3151	*Poecilochaetus*	*Terebra argus*	KVG_XX	MW007648
2239_12193	*Pseudopolydora*	*Terebra argus*	KVG_XX	MW007649
8198_12470	*Scolelepis*	*Terebra argus*	KVG_XX	MW007650
11467_2585	*Spio*	*Terebra argus*	KVG_XX	MW007651
16928_19265	*Poecilochaetus*	*Neoterebra dislocata*	Tamp_17	MW007652
15215_15537	*Scolelepis*	*Neoterebra dislocata*	Tamp_17	MW007653
11905_7897	*Spio*	*Neoterebra dislocata*	Tamp_17	MW007654
7458_6196	*Spio*	*Neoterebra dislocata*	Tamp_17	MW007655
19488_4690	*Haplosyllis*	*Neoterebra dislocata*	Tamp_18	MW007656
12507_28538	*Polydora_Dipolydora*	*Neoterebra dislocata*	Tamp_18	MW007657
15046_21361	*Scolelepis*	*Neoterebra dislocata*	Tamp_18	MW007658
5210_19368	*Spio*	*Neoterebra dislocata*	Tamp_18	MW007659
11238_14931	*Neanthes*	*Neoterebra dislocata*	Tamp_50	MW007660
10609_20576	*Poecilochaetus*	*Neoterebra dislocata*	Tamp_50	MW007661
20223_8654	*Pseudopolydora*	*Neoterebra dislocata*	Tamp_50	MW007662
7408_23902	*Scolelepis*	*Neoterebra dislocata*	Tamp_50	MW007663
22892_17291	*Spio*	*Neoterebra dislocata*	Tamp_50	MW007664

11731_11525	*Odontosyllis*	*Neoterebra dislocata*	Tamp_63	MW007665
13896_16114	*Poecilochaetus*	*Neoterebra dislocata*	Tamp_63	MW007666
29256_15611	*Scolelepis*	*Neoterebra dislocata*	Tamp_63	MW007667
26665_23868	*Spio*	*Neoterebra dislocata*	Tamp_63	MW007668
